# Living and dying with metastatic bowel cancer: Serial in‐depth interviews with patients

**DOI:** 10.1111/ecc.12653

**Published:** 2017-02-01

**Authors:** E. Carduff, M. Kendall, S.A. Murray

**Affiliations:** ^1^ Marie Curie Hospice Glasgow UK; ^2^ Primary Palliative Care Research Group The Usher Institute of Population Health Sciences and Informatics The University of Edinburgh Medical School Edinburgh UK

**Keywords:** bowel cancer, narrative, palliative care, palliative chemotherapy, qualitative longitudinal, uncertainty

## Abstract

Colorectal cancer is the second highest cause of cancer deaths. There are significant physical and psycho‐social effects on quality of life with advanced disease. Despite this, there are few accounts of the patient experience from advanced illness through to dying. We elicited the longitudinal experiences of living and dying with incurable metastatic colorectal cancer by conducting serial interviews with patients for 12 months or until they died. The interviews were analysed, using a narrative approach, longitudinally as case studies and then together. Thirty‐six interviews with 16 patients were conducted. Patients experience metastatic colorectal cancer in three phases; (1) Diagnosis and initial treatment; (2) Deterioration and social isolation and (3) Death and dying. Many patients initially said they hoped to survive, but, as “private” and in‐depth accounts of the experience emerged in further interviews, so did the understanding that this hope co‐existed with the knowledge that death was near. Palliative chemotherapy and the challenge of accessing private accounts of patient experience can inhibit care planning and prevent patients benefitting from an active holistic palliative care approach earlier in the disease trajectory. This study has immediate clinical relevance for health care professionals in oncology, palliative care and primary care.

## 1 INTRODUCTION

Colorectal cancer is the fourth most common cancer in the United Kingdom (UK), and the second highest cause of cancer death (Cancer Research UK, 2013). Bowel cancer screening has reduced the mortality rate by 27% (Libby et al., 2012), but prognosis is poor in late stage presentation (Cancer Research UK, 2013). Patients with Dukes’ stage D cancer have a 5‐year survival rate of 7% compared with 93% for those with Dukes’ A (National Cancer Intelligence Network, 2012). Colorectal cancer commonly presents with rectal bleeding, altered bowel habit and abdominal pain (Broughton, Bailey, & Linney, 2004; Cancerbackup, 2007). Patients can also experience faecal incontinence, due to abnormal bowel functioning, resulting in feeling less control over their bodies (Beech, Arber, & Faithfull, 2011; Desnoo & Faithfull, 2006; Houldin & Lewis, 2006; McCaughan, Prue, Parahoo, McIlfatrick, & McKenna, 2011). Fluctuations in psycho‐social and spiritual wellbeing also occur as patients suffer gradual social decline and seek meaning and purpose in their distress (Murray et al., 2007). Patients may also reflect on life's value as the illness progresses (Houldin & Lewis, 2006; Sjovall, Gunnars, Olsson, & Thome, 2011) and their sense of self is compromised, which can result in social isolation and existential distress (Hubbard, Kidd, & Kearney, 2010; Kidd, Kearney, O'Carroll, & Hubbard, 2008; Little, Jordens, Paul, Montgomery, & Philipson, 1998; Rozmovits & Ziebland, 2004; Taylor, 2001).

The World Health Assembly in 2014 resolved that a palliative care approach should be integrated into the care of all patients with advanced cancer (Scottish Intercollegiate Guidelines Network, 2011; World Health Assembly, 2014). However, much of the current literature is focussed on the experiences of those living with advanced cancer. Few of these studies follow participants to death to capture their longitudinal experiences before dying (Browne et al., 2011; Little et al., 1998; Ramfelt, Severinsson, & Lutzen, 2002; Sahay, Gray, & Fitch, 2000; Shaha & Cox, 2003; Sjovall et al., 2011). This has potential implications for the development of interventions to support this group of patients (Cameron & Waterworth, 2014) and is inequitable, as those with advanced disease have the greatest needs and poorer psycho‐social outcomes in terms of quality of life, anxiety, depression, social support, physical, functional and emotional wellbeing and satisfaction with medical interventions (Simon, Thompson, Flashman, & Wardle, 2008). This paper reports the longitudinal experiences, perceptions and service use of patients with metastatic colorectal cancer.

## 2 METHODS

### 2.1 Design

This qualitative study was underpinned by constructionist and narrative philosophies (Crotty, 1998; Elliott, 2005; Frank, 1995). We used serial in‐depth interviews to understand how patient experiences and needs changed as illness progressed (Kendall et al., 2015; Murray et al., 2009). Participants were interviewed over a year; each participating in up to three interviews. Based on previous longitudinal studies in lung cancer and glioma (Cavers et al., 2012; Kendall & Murray, 2005), this timeframe was considered appropriate to capture significant change in disease progression and experience. All procedures performed in studies involving human participants were in accordance with the ethical standards of the national research committee and with the 1964 Helsinki declaration and its later amendments or comparable ethical standards. Ethical approval for this study was granted by the South East Scotland research ethics committee (REC number 08/S1103/35). The Consolidated criteria for reporting qualitative research (COREQ) were used to report the study (Tong, Sainsbury, & Craig, 2007).

### 2.2 Sampling and recruitment strategy

Participants were purposively recruited from the outpatient clinic of a national centre for oncology in Scotland if they had been given a diagnosis of metastatic disease within 18 months of the start of the study. Clinical nurse specialists advised the researcher about potential participants and the consultant oncologist invited them to speak to the researcher who explained the study, provided an information sheet and offered to phone them a few days later. The researcher had no prior relationship with the participants and was introduced to the participants as a researcher. Participants were excluded if the recruiting clinician considered that there was a diagnosis of moderate to severe memory impairment, or if they were unlikely to survive for 6 months. All the participants had been told that their cancer was incurable. Participants were recruited until data saturation was reached with the analysis of the first round of interviews. Written consent was sought before the first interview and verbal consent subsequently.

### 2.3 Data collection

Where possible, interviews were conducted in the participants’ homes between November 2008 and July 2010. Two participants requested the interviews were at the recruiting centre. We were given a quiet room away from the main clinical area. All interviews were conducted by EC, a nurse, with advanced training in qualitative research methods and were audio‐recorded and transcribed verbatim. Participants were assigned a pseudonym. A narrative approach was chosen to understand the illness process through story‐telling (Hyden, 1997). Stories are commonplace in medicine, and narrative approaches are described as inclusive, whereby participants from all backgrounds can participate (Brown & Gilligan, 1992; Reissman, 1993). Participants were encouraged to tell their stories in their own terms and their own time. Topics were explored as they arose and a broad guide was used to ensure a range of issues were covered within each domain of illness experience (physical, psychological, social and spiritual). At subsequent interviews, patients were asked to expand on the issues that had arisen from the previous interview, ensuring an iterative approach,—an essential component of contemporary serial interview research (Holland, Thomson, & Henderson, 2006). For example, at the first interview, one participant described how he questioned “why me?” when he was first diagnosed. Subsequently, following his observations in the chemotherapy ward, he reasoned that cancer affects all types of people. If patients became upset, the researcher invited them to pause or terminate the interview. Detailed field notes, including observations of the physical environment, interaction with participants and reflections on the longitudinal method were recorded after every interview. Analysis of the field notes informed and contextualised the overall interview analysis.

### 2.4 Data analysis

Data were analysed at each interview point and then across time to elicit commonalities within the narratives. We used Brown and Gilligan's Voice Centred Relational Method (VCRM) (Brown & Gilligan, 1992; Mauthner & Doucet, 1998) which prioritises the participant's voice and their relationships, and encompasses what is said, and unsaid by participants. Interviews and transcripts were carefully listened to and read four times to capture in detail: (1) Overall plot of the transcript and the researcher's response to the story; (2) How the participant uses pronouns such as “I,” “you” and “one;” (3) Participants’ relationships and (4) The socio‐political and cultural context in which the story is told.

After each wave (interview 1, interview 2, interview 3) of interviewing was complete a summary analysis was written. Then the summary analyses were reviewed to consider issues that changed over time for each individual participant as a case study and then for the group as a whole, using a diagrammatical interpretation of the participants’ stories. The *X* axis represented the time, and the *Y* axis each of the four VCRM readings which not only displayed what had changed over time but also how the themes overlapped and interconnected with each other. When the diagrams for the group were completed, compared and contrasted, they highlighted that the patients’ experiences fell broadly into three stages, which we have categorised as themes; (1) diagnosis and initial treatment; (2) coping with deterioration; and (3) approaching death. The emerging findings were discussed with the multi‐disciplinary research team at regular intervals. Quotes are used to illustrate the main themes.

## 3 RESULTS

### 3.1 Sample characteristics

Table 1 presents the demographics of the patient participants, the time from diagnosis to recruitment and the timing of the interviews. The patient sample comprised of 10 men and 6 women, ranging in age from 48 to 80 years. Eleven participants presented with metastatic disease at diagnosis. The median time from diagnosis of metastatic cancer to recruitment was 9 months (range 2–15 months). All loss to follow up resulted from death. A total of 36 interviews were conducted (including 14 where the carer was present at the patent's request)—16, 13 and 7 at interview points one, two and three respectively, ranging in length from 30 to 120 min were conducted over 1 year.

**Table 1:** Patient characteristics

Participant pseudonym	Age (years)	Diagnosis (primary/metastatic)	Months from diagnosis of metastatic disease to recruitment	No. of interviews conducted	Status at study end
Ann	67	Colon/peritoneal metastases	2	3	Surviving
Brenda	60	Colon/liver metastases	6	1	Deceased
Andrew	57	Colon/liver metastases	9	3	Surviving
Brian	59	Colon/liver and lung metastases	<18 months	3	Surviving
Cath	48	Colon/liver and peritoneal metastases	15	2	Hospice, T3 abandoned
Chris	80	Colon/lung and liver metastases	13	3	Surviving
Duncan	59	Rectal/liver metastases	10	2	Deceased
Edward	74	Colon/peritoneal metastases	7	1	Deceased
Fred	73	Colon/lung and liver metastases	13	1	Deceased
Gordon	65	Colon/liver metastases	6	3	Surviving
Deirdre	66	Colon/cervical and vaginal metastases	5	2	Deceased
Harry	76	Colon/liver metastases	12	2	Deceased
Eve	62	Colon/lung and liver metastases	6	2	Deceased
Ian	65	Colon/liver metastases	2	3	Surviving
John	62	Colon/liver metastases	11	2	Deceased
Faye	55	Colon/lung and liver metastases	9	3	Surviving

### 3.2 Theme 1: Diagnosis and initial treatment

All participants narrated the lead up to the diagnosis, receiving the diagnosis and initial treatment. Some described incidences of delayed diagnosis which left them feeling fearful, abandoned and wondering if the delay could have led to more extensive disease. Those diagnosed with metastatic disease sometimes described disbelief.

I was absolutely gob smacked, I mean I knew it was there but I never expected it to be so extensive (Deirdre, 66 years, interview 1)

Participants had endured major surgery to remove the primary tumour in the bowel, then undergone chemotherapy. Surgery was sometimes described as a relief, if participants felt that something was being done. For others, it marked the beginning of a long, but hopeful road to recovery. The emergency nature of the surgery for bowel obstruction meant some participants suddenly faced their own mortality.

Die or don't. That's how bad it was, if you don't get it you could die and if you do get it you could die. So what choice do you have? (Brenda, 60 years, interview 1)

All participants had received chemotherapy, but in spite of the complications, participants described it as encouraging that something was being done.

But I kinda panicked when they stopped it (chemotherapy) because I'm saying that's getting me better you know? (Faye, 55 years, interview 1)

All patients had been told that their disease was incurable, yet many initially said they still hoped to be cured. This was attributable to treatment with chemotherapy by some. They were uncertain about how long they had left to live and what their quality of life would be. As a result of this they developed strategies to cope with uncertainty, such as developing their own parameters of illness and wellness, which they used to subjectively monitor their progress between visits to the outpatient clinic. For example, weight, and the presence or absence of pain, were subjectively monitored between appointments and was seen by participants as a tangible indicator of their progress.

I'm happy with myself because I havenae got any bleeding or anything like that. I suffer a wee bit of constipation erm but I'm no in pain (Faye, 55 years, interview 1)

Overall, participants tried to maintain as normal a life as possible by continuing to work, satisfy their hobbies and socialise with friends.

I just cope by trying to act really normal you know and just try not to think about it although you know obviously about an hour or so doesn't go past where I don't think about it but I try and just, you know. I'm at work all day and my colleagues are great (Cath, 48 years, interview 1)

However, as the cancer progressed, socialising became more difficult due to frequency of hospital appointments, bowel unpredictability, tiredness and the side effects of chemotherapy. The illness also put a strain on relationships—one participant described being well supported by her family and friends, but that no one could fully understand how she felt.

### 3.3 Theme 2: Deterioration

During the second interviews, patients spoke of disease progression, their treatment, how their social world was contracting and their thoughts about the future. In spite of the initial treatment, nearly all the participants described deterioration in their condition, such as episodes of pain. Changes to physical appearance meant the cancer was more visible to others—for example, weight changes due to cachexia or steroids, and hair loss due to chemotherapy.

Well I think sometimes when people look at you I think, and I'll maybe be wrong here, but sometimes when people know that you've sort of got cancer there, sort of, I don't know if they avoid you or they stand back (Andrew, 57 years, interview 2)

Following extensive abdominal surgery, Ann no longer felt like her old self, and she too was worried about how her physical appearance would alter her personal relationships.

You can understand how demoralising that is, and how totally disgusted I am. I can't go and visit friends like that. Even though they would understand, well would they, you know? (Ann, 67 years, interview 2)

Male, more than female participants, expressed a desire to ‘fight’ the cancer; often for the sake of others. Some took hope from previous experiences to fuel their positive outlook. For example, Gordon was encouraged that in the past he had been given good news at the clinic, so he expected it again. John had already survived 18 months which he considered a positive sign.

I mean, I've survived 18 months plus, because there's the time before when I had the cancer and I didn't know about it. So I mean, I'm 18 months down the line and I didn't think I'd be 18 months down the line to be honest with you. I mean, when somebody tells you you've got cancer you think “oh shit” you know – excuse my French! (John, 62 years, interview 2)

Treatment played a vital role as it enabled participants to feel that they were being actively cared for, as opposed to feeling abandoned. In spite of their incurable status, treatment brought hope, as the quote from Andrew illustrates.

the doctor said there would be other drugs, if it was necessary we could use them as well. But the chemo itself seems to be shrinking it (Andrew, 57 years, interview 2)

Maintaining this positive exterior was difficult, and participants expressed that the thought of dying was at the back of their minds.

when they [family] all go away, you can come down because you're being so positive. But there's also that nagging at the back of your head that it's not. (Faye, 55 years, interview 2)

Existential aspects of the experience became more obvious at interview 2, as many participants were consumed by thoughts of what the future would hold for them.

I try not to think about it anymore than I can help. I mean, it's generally in my bed at night and my brain starts whirling, sometimes in the morning as well when I wake up (Deirdre, 66 years, interview 2)

Akin to the findings in the first interviews, participants’ thoughts of the future were veiled by feelings of uncertainty. Although each had been told the cancer was not curable, participants were uncertain about how the illness would progress and what their future would hold, even hoping they may avoid their likely fate. However, some participants had started planning for dying with their relatives.

And we were talking about that, my sister and I, I says ‘well, to be honest, I'm not wanting that [to die at home] for anybody’ I says ‘I'd just go into the hospital’ (Faye, 55 years, interview 2)

Participants expressed concern about what material and an emotional legacy they were leaving their families. Many participants wanted to know that their lives had been meaningful. For example, Duncan wanted to be remembered fondly by people.

I mean, the most important thing is for people to turn around when it's your time to move on and they say ‘well, your dad was a gentleman’ (Duncan, 57 years, interview 2)

### 3.4 Theme 3: Last months of life

At the time of the third interviews, most participants were more openly acknowledging their days were numbered. Considerable physical deterioration was reported and existential issues were more readily expressed. Participants spoke of death with more certainty. Home became a more meaningful place, where family, friends and memories were kept alive. Several participants spoke of dying at home in a familiar and secure place. Chris wished to stay at home providing his wife could cope.

If it's not going to be too much for Hazel, that's the only thing. If it's going to be too much for her I'll just go into the hospital. But she's coping alright. (Chris, 80 years, interview 3)

Physical deterioration brought increasing weakness, dependency and social isolation. Participants expressed a desire to maintain a semblance of normality in their lives, but illness progression made this increasingly difficult. Patients spoke about their inability to socialise, maintain old and develop new relationships. They worried about becoming a burden on their family and friends. Many described losing confidence in their bodies, but treatment with chemotherapy (which was universally palliative) or a possible new treatment discovery were considered the only lifelines and enabled some participants to remain falsely hopeful of survival.

they [health professionals] also said at the time “there might be something that comes along, you never know” so therefore that's in the back of your mind as well (Andrew, 57 years, interview 3)

Waiting to be told that death was imminent was consuming the attention of many participants. Some were imminently dying, and palliative care had been initiated to manage the last few weeks at home. However, Ian had been given a 2‐year prognosis at the time of his diagnosis, so as far as he was concerned, he was approaching the end when he reached 18 months. In comparison, Andrew had not been given a prognosis in terms of months or years, but had devised his own strategy for dealing with the uncertainty of his future by setting goals of things he wanted to achieve or events he wanted to attend.

## 4 DISCUSSION

### 4.1 Summary

Patients with metastatic colorectal cancer have complex progressive physical symptoms, substantial treatment burdens and multi‐dimensional needs. They also experience progressive psychological, social and existential challenges. Their experience was similar to that previously described in patients with advanced lung and brain cancer (Cavers et al., 2012; Kendall & Murray, 2005) in that there was a clear beginning to the story, and contrasted to narratives of patients with chronic obstructive pulmonary disease and heart failure (Kendall et al., 2015; Murray et al., 2007; Pinnock et al., 2011). An understanding of the difference between public and private accounts which patients tell clinicians, and the importance of current social narratives of cancer, was vital for understanding the results of this study.

Over time the psycho‐social and spiritual aspects of the illness played a significant role, as people sought meaning in life and relationships (Simon et al., 2008). Participants strived to maintain their sense of self through diagnosis, as they deteriorated, and through death. Consistent with the findings of other studies, at diagnosis, participants tried to make sense of, and accommodate the cancer in their lives (Ramfelt et al., 2002) and maintain a semblance of normality (Taylor, Richardson, & Cowley, 2010), but over time became increasingly isolated and their social world contracted (Little et al., 1998; Taylor, 2001).

Narratives of uncertainty dominated the accounts, despite being told that their cancer was incurable and death inevitable. This supports previous reports describing uncertainty throughout the illness journey and in relation to the future, families, treatment, coping mechanisms, worsening illness (Beaver et al., 2010; Browne et al., 2011; McCaughan et al., 2011; Shaha, Cox, Talman, & Kelly, 2008; Taylor et al., 2010). Feeling uncertain is common to the experience of many diseases (Etkind, Bristow, Bailey, Selman, & Murtagh, 2016; Kimbell, Boyd, Kendall, Iredale, & Murray, 2015; Taylor, Wells, Hubbard, & Worth, 2016). Recent research has shown that uncertainty is related to the participants’ engagement with the illness process, that is, the extent to which they take control or cede control to professionals and their level of understanding of what is often complex disease trajectory (Etkind et al., 2016). In our study, uncertainty was expressed increasingly, over time, as the impact of feeling uncertain, yet certain of death, emerged. This competing story of an uncertain prognosis with a certain outcome, which developed over time, left patients feeling “betwixt and between” (Hockey, 2002; Van Gennep, 1960).

The findings suggest that participants did know they were dying, but this was not necessarily expressed until the later interviews. This contrasts with recent research that reported 81% of patients with colorectal cancer hoped that their palliative chemotherapy was going to cure them (Weeks et al., 2012). The high number in this, and some other studies, may be due to patients expressing their “public accounts,” while our serial in‐depth interviews managed to access their private accounts.

This study identifies potential explanations why the public narrative may be often told by patients and captured in quantitative research. Treatment with palliative chemotherapy can be understood by patients as a possible lifeline. This hopeful, but unrealistic, narrative is taken up and continued by the clinician, as it is easier to have a hopeful conversation, than take time to explore the more complex, existential challenges that the participants described. We found that participants did not necessarily speak of palliative chemotherapy extending their lives or managing their symptoms in the interviews, but ending treatment was seen as a sign that health professionals had given up on them.

The narrating, and listening, to idealised public accounts of illness can be potentially problematic. It promotes survivorship, of ‘fighting’ cancer. This “folie a doux” inhibits patients and professionals discussing early palliative care and advance care planning (Wright, Zhang, Keating, Weeks, & Prigerson, 2014).

### 4.2 Implications

There are implications for oncologists, palliative care specialists and generalists in terms of communication and advance care planning. Health professionals need to manage uncertainty through the provision of information, including that the prognosis is often unclear. Managing expectations and helping those with colorectal cancer to plan for the future may reduce feelings of doubt and insecurity towards the end of life. The findings of this research highlight the potential of multi‐disciplinary involvement, including palliative care teams, and good communication about the intent and limits of treatment, which have been discussed with the patient. Ending palliative chemotherapy is a critical time on the illness trajectory of people with cancer, and active support should be triggered, if not already in place. The offer of ongoing care and support during and following chemotherapy may allow a realistic transition to discussing goals of care, and allow care planning to be more patient centred. Currently, palliative care is not always seen as an active and alternative treatment option, rather as planning for dying, which is an unattractive option for patients. Changing this will enable patients to simultaneously receive chemotherapy and palliative care.

### 4.3 Strengths and limitations

We could find no other serial in‐depth interview studies of patients dying with metastatic colorectal cancer. The longitudinal approach permitted the researcher to follow participants as they deteriorated, so those in the last months of life were represented. The design enabled prospective, retrospective and real‐time accounts of experience, where participants moved beyond public accounts of illness to reveal their private thoughts and fears (Cornwell, 1984; Murray et al., 2009). Therefore, the study was not based solely on retrospective accounts of experience. No participants withdrew from the study, which suggests that the mode of recruitment and the longitudinal method was acceptable to participants. Regular research team meetings were conducted to ensure adequate reflection time and to consider the boundaries of the researcher/participant relationship. Managing and analysing the large dataset was complex. It is for this reason that there is a drive to promote secondary analysis of the rich, qualitative data which arise from longitudinal work. The data generated from this study continues to be used in this way (Kendall et al., 2015).

This study affords a rich, contextualised account of the experiences of this group of participants. Different accounts may have been heard had different participants been recruited, at a different time, and from a different place, or ethnic of cultural grouping. Also, those with a permanent stoma were under‐represented. However, the generalisability to other populations is best considered by comparing the contexts (Polit & Beck, 2010). Patients were not asked about which aspect of care they might prioritise, so a further study is indicated before a pilot intervention may be tested to do this.

## 5 CONCLUSIONS

Living and dying with advanced colorectal cancer is multi‐faceted; patients have in their minds contradictory accounts of certainty and uncertainty, recovery and death, hope and despair. Patients express one or other of these to clinicians and researchers according to how safe or secure they may feel and their relationship with the enquirer. Good communication, the provision of information and shared decision making are important to help reduce feelings of anxiety and to enable patients to voice their fears, and prepare for the future through and after their deaths. A palliative care approach, which encourages holistic care to enable those who are dying to maintain a sense of meaning and purpose, should be integrated into oncological and primary care from diagnosis of advanced disease.

## CONFLICTS OF INTEREST

The authors declare that they have no conflict of interest.

## ACKNOWLEDGEMENTS

This work was supported by a PhD studentship from the Economic and Social Research Council. We would like to offer our gratitude to those who participated in this study and to the staff at the recruiting centre.
